# Modulation of Force below 1 Hz: Age-Associated Differences and the Effect of Magnified Visual Feedback

**DOI:** 10.1371/journal.pone.0055970

**Published:** 2013-02-11

**Authors:** Emily J. Fox, Harsimran S. Baweja, Changki Kim, Deanna M. Kennedy, David E. Vaillancourt, Evangelos A. Christou

**Affiliations:** Department of Applied Physiology and Kinesiology, University of Florida, Gainesville, Florida, United States of America; University of Alberta, Canada

## Abstract

Oscillations in force output change in specific frequency bins and have important implications for understanding aging and pathological motor control. Although previous studies have demonstrated that oscillations from 0–1 Hz can be influenced by aging and visuomotor processing, these studies have averaged power within this bandwidth and not examined power in specific frequencies below 1 Hz. The purpose was to determine whether a differential modulation of force below 1 Hz contributes to changes in force control related to manipulation of visual feedback and aging. Ten young adults (25±4 yrs, 5 men) and ten older adults (71±5 yrs, 4 men) were instructed to accurately match a target force at 2% of their maximal isometric force for 35 s with abduction of the index finger. Visual feedback was manipulated by changing the visual angle (0.05°, 0.5°, 1.5°) or removing it after 15 s. Modulation of force below 1 Hz was quantified by examining the absolute and normalized power in seven frequency bins. Removal of visual feedback increased normalized power from 0–0.33 Hz and decreased normalized power from 0.66–1.0 Hz. In contrast, magnification of visual feedback (visual angles of 0.5° and 1.5°) decreased normalized power from 0–0.16 Hz and increased normalized power from 0.66–1.0 Hz. Older adults demonstrated a greater increase in the variability of force with magnification of visual feedback compared with young adults (P = 0.05). Furthermore, older adults exhibited differential force modulation of frequencies below 1 Hz compared with young adults (P<0.05). Specifically, older adults exhibited greater normalized power from 0–0.16 Hz and lesser normalized power from 0.66–0.83 Hz. The changes in force modulation predicted the changes in the variability of force with magnification of visual feedback (R^2^ = 0.80). Our findings indicate that force oscillations below 1 Hz are associated with force control and are modified by aging and visual feedback.

## Introduction

Oscillations in motor output change in specific frequency bins and have important implications for understanding healthy aging [1], [2], [3], [4] and pathological motor control [5], [6], [7]. Constant force contractions, which comprise primarily of low-frequency oscillations [4], [8], [9], [10], often are used as a model to understand the impaired force control in older adults and the associated physiological mechanisms (for review see Enoka et al. 2003). The age-associated differences in force control occur primarily at very low-force levels [2], [11] and are functionally relevant for many activities of daily living (e.g. writing, buttoning a shirt, manipulating objects) [12], [13]. In this study we focused on low-frequency oscillations in force because they have been associated with impaired submaximal force control in older adults.

Low-frequency oscillations in force have been examined grossly, as evidenced by prior studies that report average power across a broad bin (e.g. 0–4 Hz) [3], [4], [14]. Recent findings from our lab, however, demonstrate that the modulation of force during constant force contractions occurs primarily from 0–1 Hz [8]. Furthermore, the greatest differences in force spectra for young and older adults occur from 0–1 Hz [4], [14], which suggests that modulation from 0–1 Hz is particularly relevant to understanding age-related impairments in force control. In addition, the age-associated differences in force control are exacerbated when visual feedback is magnified [15], [16], which also influences the power from 0–1 Hz. Specifically, magnification of visual feedback increases power from 0–1 Hz [17], [18], whereas removal of visual feedback decreases power from 0–1 Hz [3], [9]. Because these prior studies averaged power across the 0–1 Hz bin they have assumed that force is modulated uniformly in all the frequencies below 1 Hz. However, specific force frequencies may be modulated differently in response to aging and visual feedback.

While substantial evidence indicates that modulation of force from 0–1 Hz is influenced by aging and visuomotor processes, specific force oscillations *below* 1 Hz may be associated with distinct physiological processes. These slow oscillating force signals, however, have not been examined. Low-frequency oscillations below 1 Hz have been recorded in axial and limb muscles during sleep and awake periods [19]. These oscillations, which occurred at 0.05 and 0.2 Hz, were not associated with cardiac rhythms and were thought to originate from cortical rhythms. Indeed, oscillations below 1 Hz are evident in electroencephalogram recordings [20] and intercellular recordings from several cortical structures [21]. Respiratory control may be another source of low-frequency oscillations [22] and we have recently demonstrated that respiration and visual feedback interact and alter the modulation of force [17].

Our previous findings from this dataset demonstrated that older adults compared with young adults exhibit impaired force control with magnified visual feedback. This study, however, did not examine specific frequencies below 0–1 Hz. In this follow up study, we re-examined the interactive effects of aging and visual feedback on force control by investigating the modulation of force in specific frequencies below 1 Hz [15]. Examining force oscillations below 1 Hz allows us to determine whether this bin is modulated uniformly or whether specific frequencies below 1 Hz contribute to the age-associated differences in force control. Specifically, the purpose of this follow-up investigation was to determine whether a differential modulation of force below 1 Hz contributes to changes in force control related to manipulation of visual feedback and aging. Examining force oscillations below 1 Hz may have important implications for understanding healthy aging [11], [23], [24] and consequently pathological motor control [25]. Part of the findings has been reported in a previous paper [15] and abstract form [26].

## Methods

Ten young adults (25±4 yrs, 5 men) and ten older adults (71±5 yrs, 4 men) volunteered to participate in this study. All subjects reported that they were healthy and moderately active. They were approved by their physician to participate, demonstrating no symptoms of cardiovascular or neurological disorders. Subjects were screened for cognitive impairments and scored greater than 26 out of 30 on the Mini-Mental State Examination [27], were right-handed according to a standardized survey [28], and had normal or corrected vision.

### Ethics

The Institutional Review Board at Texas A&M University approved the procedures, and subjects provided written informed consent to participate.

### Experimental Arrangement

Subjects were seated comfortably, facing a 27-inch computer screen (Samsung Syncmaster ™ 275T+, Samsung Electronics America, NJ, USA) with a resolution of 1920×1200 pixels, which was located 1.25 m away at eye level. The monitor displayed a line representing the force produced by the abduction of the index finger. All subjects affirmed that they could see the display clearly. Some subjects wore corrective lenses. The left arm was supported in a position with the shoulder abducted approximately 45° and the elbow flexed to ∼90°. The left forearm was pronated and secured in specialized padding (Versa Form ™, AB Germa, Sweden). The left hand (non-dominant hand) was secured and stabilized on a customizable metal plate such that movement of the thumb, middle, ring, and little fingers was restricted and there was approximately a right angle between the index finger and thumb. Only the left index finger was free to move. The left index finger was placed in an adjustable finger orthosis to maintain extension of the middle and distal interphalangeal joints [29]. This arrangement allowed abduction of the index finger about the metacarpophalangeal joint in the horizontal plane, a movement produced almost exclusively by the first dorsal interosseus (FDI) muscle [30], [31]. We examined the left hand because it was the non-dominant hand and thus the task would potentially be less influenced by previous experience. In addition, most of the previous studies on abduction of the index finger were performed on the non-dominant hand (for a review see Enoka et al. 2003).

### Force Measurement

The constant isometric force produced by the abduction of the index finger was recorded with a one-dimensional force transducer (Futek LRF400 (L2338) Futek Advanced Sensor Technology Inc. CA, USA). The force signal was sampled at 1 kHz with a Power 1401 A/D board (Cambridge Electronic Design, UK) and stored on a personal computer.

### Experimental Procedures

Subjects participated in one experimental session that lasted approximately 2 hours. Each subject began with familiarization of the experimental procedures. After the familiarization, each subject performed the following: 1) MVC with abduction of the index finger; 2) Constant force task with the index finger; 3) repetition of the MVC task. For the constant force task, each subject performed three trials at each visual angle (0.05°, 0.5°, 1.5°) at 2% MVC, which resulted in 9 trials per visual feedback condition (vision and no vision). We counter balanced the order for the visual feedback conditions. For both of the visual feedback conditions, the order for the three visual angles (0.05°, 0.5°, 1.5°) was presented randomly to the subjects. This force was chosen because age-associated differences in force control are consistently evident at low-force levels (2% MVC) [32].

### Visual Feedback Manipulation

We altered the gain of visual feedback by manipulating the visual angle [15], [33]. We used the following formula to manipulate the visual angle:
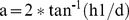
(1)where a = visual angle, h1 = ½ of the height of character (force fluctuations viewed on the screen) and d = distance of the eye to the computer screen. For each subject, the distance from the eye to the screen (d) was held constant and subjects were closely monitored to ensure that they maintained their position in the chair. To alter the visual angle, the amplitude of the force fluctuations (h1) viewed by the subject on the screen was manipulated. Based on previous studies [9], [29], [34], the amplitude of force fluctuations was estimated to be 3% of targeted force (CV of mean force).

### MVC Task

Subjects were instructed to increase the force of their left index finger abduction from baseline to maximum over a two-second period and to maintain their maximal force for 4 to 7 seconds. Trials were performed (up to five trials) until two of the maximal trials were within 5% of each other. The force produced was represented as a blue tracing and was displayed on the computer monitor to provide visual feedback. The MVC force was quantified as the average force maintained for 3–6 seconds during the trial with the highest force. This procedure allows for the identification of a more conservative MVC that reflects the capacity to maintain an isometric contraction.

### Constant Isometric Force Task

**Figure 1:**
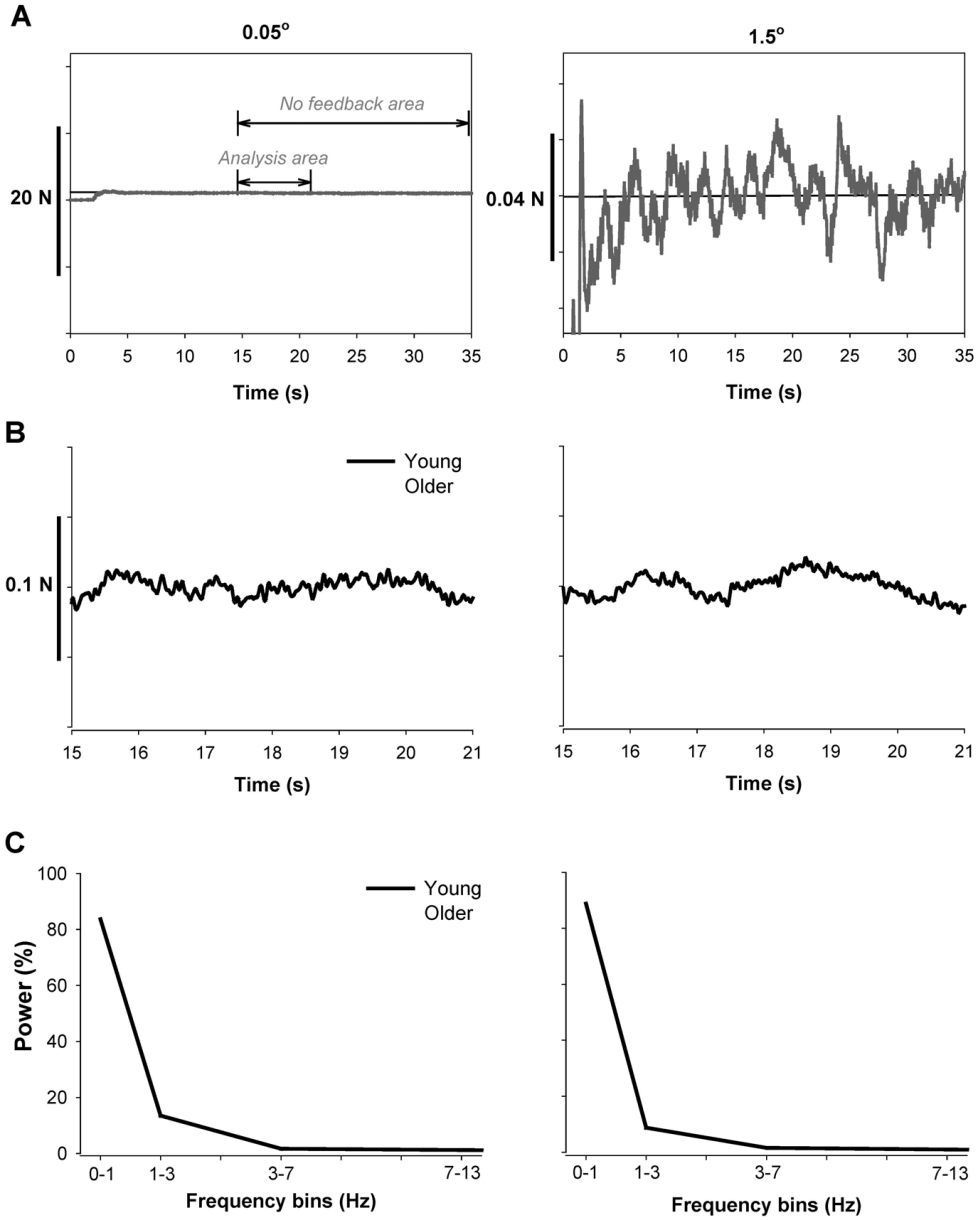
Representative trials from a young and an older adult exerting a constant force at 2% MVC with low (visual angle = 0.05°, left column) and high (visual angle = 1.5°, right column) magnification of visual feedback. A: Each subject performed a constant isometric contraction by abducting their left index finger against a force transducer. Subjects were instructed to match a line representing their force to a horizontal target line for 35 s. Visual feedback of the target line and exerted force was provided throughout the entire trial during visual feedback conditions. During no visual feedback trials, feedback was removed (no feedback area) after 15 s. B: Representative force output from a young and an older adult is shown. Data analysis was based on force output from 15–21 s (analysis area). Older adults exhibited greater variability of force with higher magnification of visual feedback (higher visual angles of 0.5° and 1.5°). C: Representative normalized power spectrum of the force output from 0–13 Hz in a young and an older adult during trials with low and high magnification of visual feedback. The majority of the power (∼85%) in the force output occurs from 0–1 Hz.

We manipulated the visual feedback condition (presence or absence of visual feedback), and the magnification of the visual feedback with a custom-written program in Matlab® (Math Works™ Inc., Natick, Massachusetts, USA). We achieved the specified visual angles (0.05°, 0.5°, 1.5°) by changing the ordinate scale, which altered the amplitude of the force fluctuations viewed by the subject on the screen (described above). A decrease in the size of the ordinate scale (zoom in) magnified the visual feedback of the force fluctuations (Figure 1A). Force fluctuations were magnified, therefore, with visual angle. The target force was indicated by a red horizontal line in the middle of the monitor and the force exerted by each subject was represented as a blue line, which progressed with time from left to right (see Figure 1 in Kennedy and Christou 2011). During the task, subjects maintained their position in the chair and were instructed to gradually push against the force transducer and increase their force (blue line on the monitor) to match the target force (red line) within 5 seconds. When the target was reached, subjects were instructed to maintain their force on the target as accurately and consistently as possible. Each trial lasted 35 s.

During the visual feedback condition, subjects viewed both their force and target lines during the entire trial (Figure 1A). During the no visual feedback condition, subjects viewed their force and target lines for the first 15 s, after which visual feedback was removed (Figure 1A). The rest time between visual feedback conditions and visual angle trials was 30 s. The rest time between each trial within visual feedback conditions and visual angles was 15 s.

### Data Analysis

Data were acquired with the Spike2 software (Version 6.02; Cambridge Electronic Design, Cambridge, UK) and analysed off-line using custom-written programs in Matlab® (Math Works™ Inc., Natick, Massachusetts, USA). For both vision conditions, 6 s of force data were used in the analyses (Figure 1; also see Figure 1, Kennedy and Christou 2011). We used 6 s of data to minimize the influence of the drift on the force signal. The force signal was filtered with a 4^th^-order (bi-directional) Butterworth filter using a 20 Hz low-pass cut-off. The coefficient of variation of force (CV) was quantified from the detrended force output to minimize the effect of any drift from the targeted force (especially during the absence of visual feedback condition) on force variability. This was achieved by removing the linear trend from the force data. The dependent variables were the mean force, standard deviation (SD) of force, the coefficient of variation of force (CV; (SD of force/mean force)×100), and the change in CV of force. The independent variables were age of the subjects (young and older), visual feedback condition (vision and no vision), and visual angle (0.05°, 0.5°, 1.5°). For the no visual feedback condition we used the no visual feedback data that was preceded by visual feedback with the lowest visual angle. We selected these data because the no visual feedback condition (preceded by the lowest visual angle) was least likely to be influenced by the visual feedback condition that preceded it.

### Power Spectrum of Force

A Fourier analysis was performed on the force signal [35]. The sampling frequency was 1 kHz. The window size was 6000, which gave a resolution of 0.166 Hz. For statistical comparisons, the frequency data of the force signal were divided into seven frequency bins: 0 (0–0.08), 0.16 (0.09–0.24), 0.33 (0.25–0.41), 0.50 (0.42–0.58), 0.66 (0.59–0.72), 0.83 (0.73–0.91), and 1.00 (0.92–1.08) Hz. These frequency bins, therefore, were based on the highest resolution of the Fourier analysis that could be accomplished with 6 s of force data. The dependent variables for the spectral analysis of the force signal were the absolute (N^2^) and normalized power (%) in each data bin. We examined both absolute and normalized power of the force spectra to fully evaluate the changes in force oscillations in response to the manipulations performed (aging and visual feedback). The absolute power is influenced by the amplitude of force variability, whereas the relative power reflects the changes in the structure of the force signal independent of force variability. The normalized power was calculated as the absolute power in each frequency bin relative to the total power of the force signal from 0–1 Hz. Henceforth, the normalized power also is referred to as relative power.

### Statistical Analysis

Analyses were performed with the PASW Statistics 18.0 statistical package (SPSS Inc., Chicago, IL). To determine the change in CV of force relative to the no visual feedback condition for young and older adults we used a mixed ANOVA (2 age groups×3 visual angles) with repeated measures on visual angles. To determine the effect of removal of visual feedback on absolute and relative power in frequencies below 1 Hz for young and older adults we used mixed ANOVAs (2 visual conditions×7 frequency bins) with repeated measures on visual feedback condition and frequency bins. Finally, to determine the interactive effects of aging and amount of visual feedback we used mixed ANOVAs (2 age groups×3 visual angles×2 visual feedback conditions×7 frequency bins) with repeated measures on visual angle (0.05°, 0.5° and 1.5°), visual feedback condition (vision, no vision) and frequency bins (seven frequency bins: 0, 0.16, 0.33, 0.50, 0.66, 0.83, and 1.0 Hz). When Mauchley’s test indicated that the assumption of sphericity was violated the degrees of freedom were corrected using Huynh-Feldt estimates of sphericity.

Significant interactions from the ANOVA models were followed by appropriate post hoc analyses. For example, age-associated differences were followed with independent t-tests, whereas differences between visual feedback conditions were examined with paired t-tests. Multiple t-test comparisons were assessed using the Tukey HSD (honestly significant differences) test.

Backward multiple linear regression models were used to establish a statistical model that predicted the change in CV of force (criterion variables) from the change in power (absolute power as well as relative power) of the seven frequency bins (predictor variables). The change in CV of force was quantified from the CV of force during the highest visual angle relative to the no visual feedback condition. The backward model was accepted if all predictor variables significantly contributed to the criterion variable. The goodness-of-fit of the model, which indicates how well the linear combination of the variables predicted the change in the CV of force, was given by the squared multiple correlation (R^2^) and the adjusted squared multiple correlation (adjusted R^2^). The adjusted R^2^ is reported because the R^2^ can overestimate the percentage of the variance in the criterion variable that can be accounted for by the linear combination of the predictor variables, especially when the sample size is small and the number of predictors is large [36].

Unless corrected, the alpha level for all statistical tests was 0.05. Data are reported as means ± standard error of the mean.

## Results

### Strength, Fatigue, and Force Variability

**Figure 2:**
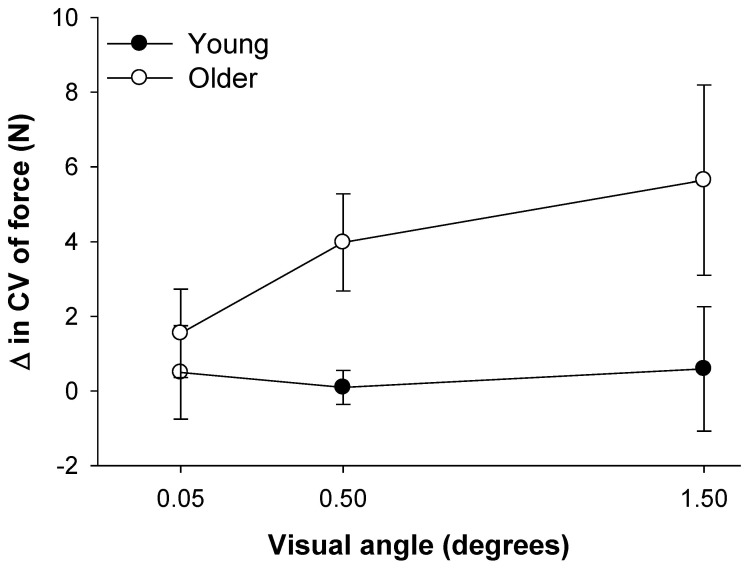
The change in CV of force with magnification of visual feedback for young and older adults. The change in CV of force at each visual angle was calculated relative to the no visual feedback condition. On average, older adults exhibited significantly greater change in the CV of force across all visual angles. The greatest age differences in the change of the CV of force occurred at the moderate and high visual angles.

During this experiment young and older adults exhibited similar MVC before (young: 25.0±12.0 N; older: 25.0±4.1 N; P>0.5) and after (young: 24.8±10.7 N; older: 25.3±4.7 N; P>0.5) the experimental session [15]. This indicates that the experimental protocol did not induce any fatigue in our subjects. Furthermore, as we have previously reported [15], older adults exhibited greater CV of force compared with young adults, especially during the highest visual angle condition (young vs. older; 0.05°: 5.62±1.01 vs. 6.30±1.34; 1.5°: 5.59±1.17 vs. 9.03±2.48). For this study, we focused on low-force contractions (2% MVC) and examined the change in CV of force for each visual angle relative to no visual feedback condition. On average, older adults exhibited greater change in the CV of force than young adults (F_1,18_ = 4.3, P = 0.05). Based on the pattern of data, which is evident from Figure 2, the age-associated differences in the change of CV of force were greater for the largest visual angle (1.5°). Thus, because the protocol did not induce fatigue in the subjects, these findings indicate that the differences in the variability of force were independent of the subjects’ strength. Furthermore, these findings suggest that magnification of visual feedback exacerbated the age associated differences in the variability of force (Figures 1 and 2).

### The Effect of Removal of Visual Feedback

**Figure 3:**
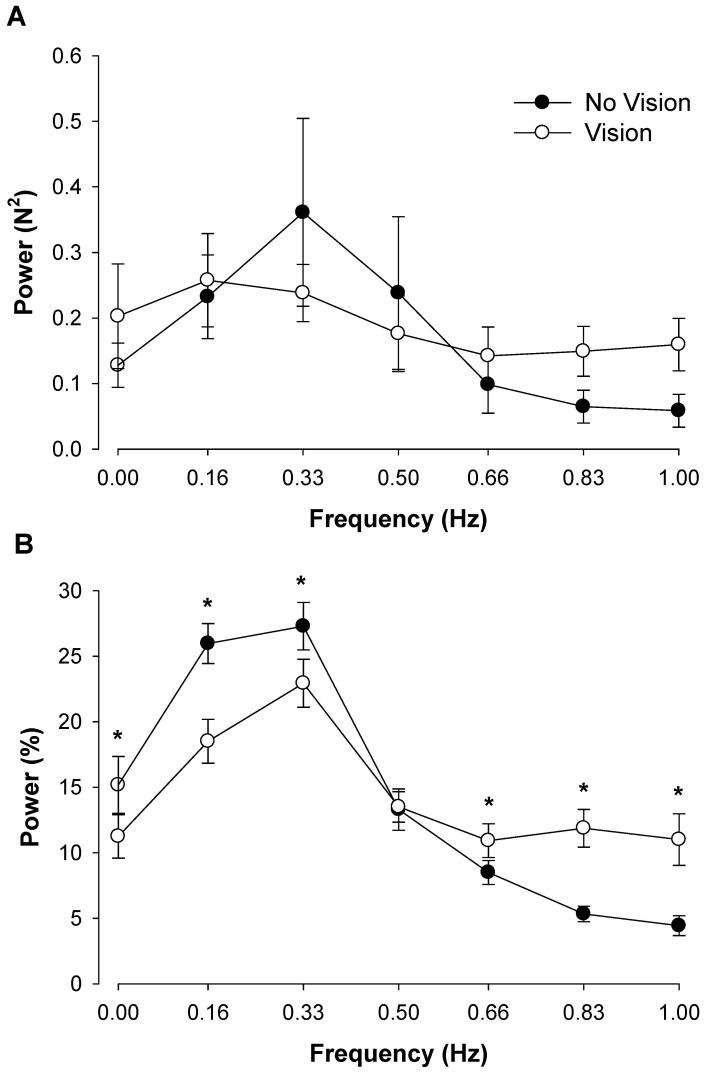
The effect of no visual feedback on force oscillations below 1 Hz. A: The absolute power during the two visual feedback conditions. The age and visual feedback condition main effects and associated interactions were not significant. B: The normalized power during the two visual feedback conditions. During the no visual feedback condition, normalized power in the force output at 0.16 Hz increased; whereas normalized power decreased from 0.83–1.0 Hz. Young and older adults exhibited a differential modulation of force oscillations below 1 Hz during the no visual feedback condition compared with visual feedback at the highest visual angle (1.5°). Asterisk (*) indicates significant difference (P<0.05) between the no visual feedback condition and the visual feedback condition.

To determine whether modulation of force output below 1 Hz is different with and without visual feedback we compared the two extreme conditions. Specifically, we compared the no visual feedback condition preceded by the lowest visual feedback angle (0.05°) with the visual feedback condition at the highest visual angle (1.5°). For the absolute power spectrum there was a significant frequency main effect (F_6,84_ = 3.7, P<0.01). The interaction between visual feedback condition and frequency was not significant (P>0.1; Figure 3A). Nonetheless, inspection of the data (Figure 3A) suggests that during the no visual feedback condition subjects exhibited greater power at 0.33 Hz and lesser power at 1.0 Hz. For the normalized power spectrum, there was a significant visual feedback condition×frequency interaction (F_6,114_ = 4.65, P<0.001; Figure 3B). Visual inspection of the interaction indicated that during the no visual feedback conditions, both groups exhibited greater relative power from 0 to 0.33 Hz and lesser relative power from 0.66 to 1.0 Hz. Post hoc analyses indicated that during the no visual feedback conditions, power was higher at 0.16 Hz (|t_19_| = 2.7, P<0.008) and lower from 0.83 to 1.0 Hz (|t_19_|>3.0, P<0.004).

### The Effect of Magnification of Visual Feedback

**Figure 4:**
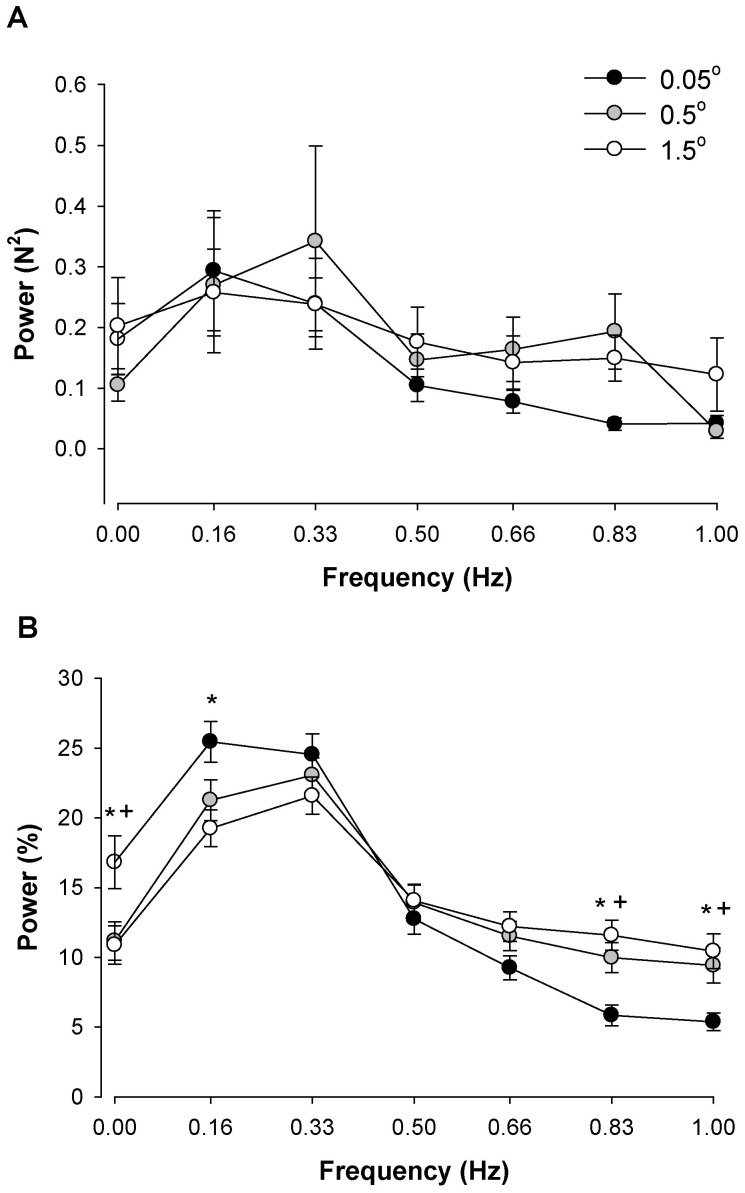
The effect of visual angle and frequency bins below 1 Hz. A: The absolute power as a function of frequency bins during the three different visual angles. The age and visual angle main effects and associated interactions were not significant. B: The normalized power during the two visual feedback conditions. Magnification of visual feedback at the highest visual angle (1.5°) compared with the lowest visual angle (0.05°) significantly decreased force oscillations from 0–0.16 Hz and increased power from 0.83–1.0 Hz. Visual feedback at a moderate visual angle (0.5°) compared with the lowest visual angle (0.05°) increased power at 0 Hz and decreased power from 0.83–1.0 Hz. Asterisk (*) indicates significant difference (P<0.05) between the highest (1.5°) and lowest (0.05°) visual angle. A cross (+) indicates significant difference (P<0.05) between the moderate (0.5°) and lowest (0.05°) visual angle.

For the absolute power spectrum there was a significant frequency main effect (F_6,72_ = 4.3, P<0.01). The interaction between visual feedback angle and frequency was not significant (P = 0.69; Figure 4A). Visual inspection of the data demonstrated that magnification of visual feedback increased absolute power from 0.66 to 0.83 Hz. For the normalized power spectrum there was a significant visual feedback angle×frequency interaction (F_12,216_ = 3.79, P<0.001; Figure 4B). Based on visual inspection of this interaction, magnification of visual feedback decreased relative power from 0 to 0.16 Hz and increased relative power from 0.66 to 1.0 Hz. Post hoc analyses (Tukey HSD) demonstrated that when subjects received visual feedback at the highest visual angles (0.5° and 1.5°) compared with the lowest visual angle (0.05°) they decreased power in their force output from 0–0.16 Hz (P<0.05) and increased power from 0.83 to 1.0 Hz (P<0.05).

### The Effect of Age and Visual Feedback

**Figure 5:**
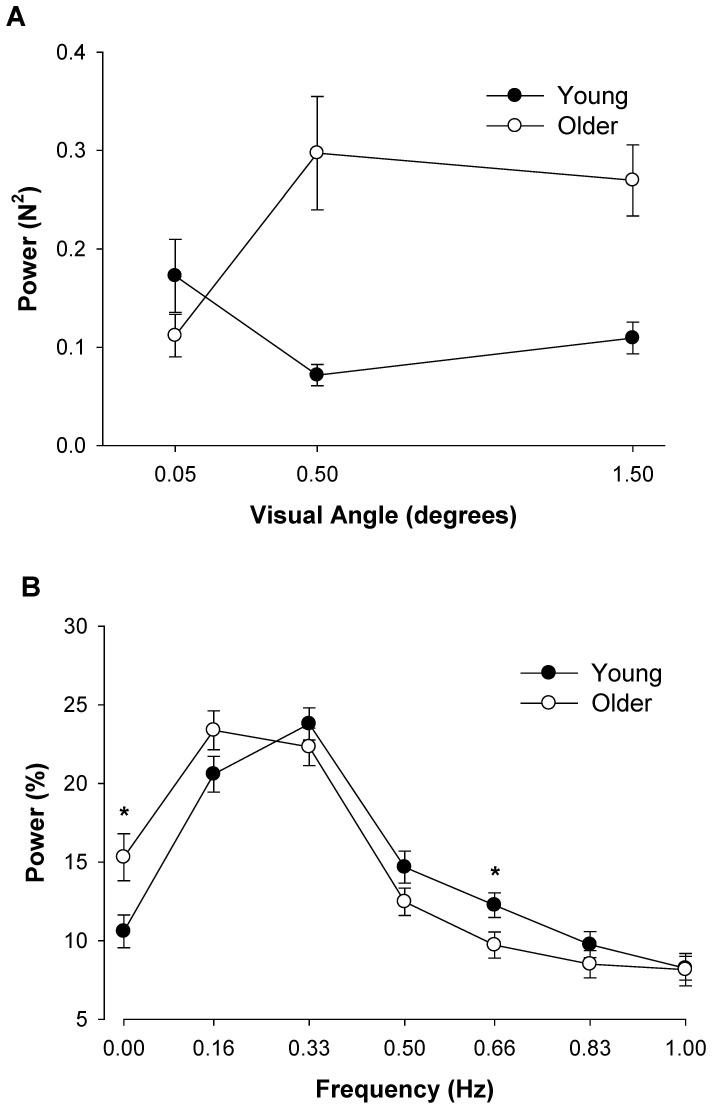
The interaction of age and frequency bins below 1 Hz. A: The absolute power during the three different visual angles. The age×visual angle approached significance (P = 0.06) and suggests that power within 0–1 Hz was greater in older adults for the visual angles that magnified the visual feedback. B: The normalized power for young and older adults at different frequency bins. On average, compared with young adults, older adults exhibited greater normalized power from 0–0.16 Hz and lower normalized power from 0.5–0.83 Hz. Post hoc analysis, indicates that these differences were statistically significant (P<0.05) between young and older adults at 0 Hz and 0.66 Hz. Asterisk (*) indicates significant difference (P<0.05) between young and older adults.

For the absolute power spectrum, the age×visual feedback angle interaction approached significance (F_2,24_ = 3.1, P = 0.06; Figure 5A). Visual inspection of the data demonstrated that magnification of visual feedback increased power below 1 Hz in older adults and decreased power below 1 Hz for young adults. The age main effect (P = 0.68) and the age×frequency bin interaction were not significant (P = 0.82). For the normalized power spectrum of force, there was a significant age×frequency bin interaction (F_6, 108_ = 2.59, P<0.05; Figure 5B). The interaction indicated that older adults compared with young adults exhibited greater relative power from 0 to 0.16 Hz and lesser relative power from 0.66 to 0.83 Hz under all visual feedback conditions. Post hoc analyses indicated that older adults compared with young adults exhibited greater power at 0 Hz (|t_18_| = 2.6, P<0.01) 0.16 Hz (|t_18_| = 1.5, P = 0.07) and lesser power from 0.66 Hz (|t_18_|>1.9, P<0.05).

### Prediction of the Change in Force Variability with Magnification of Visual Feedback

**Figure 6:**
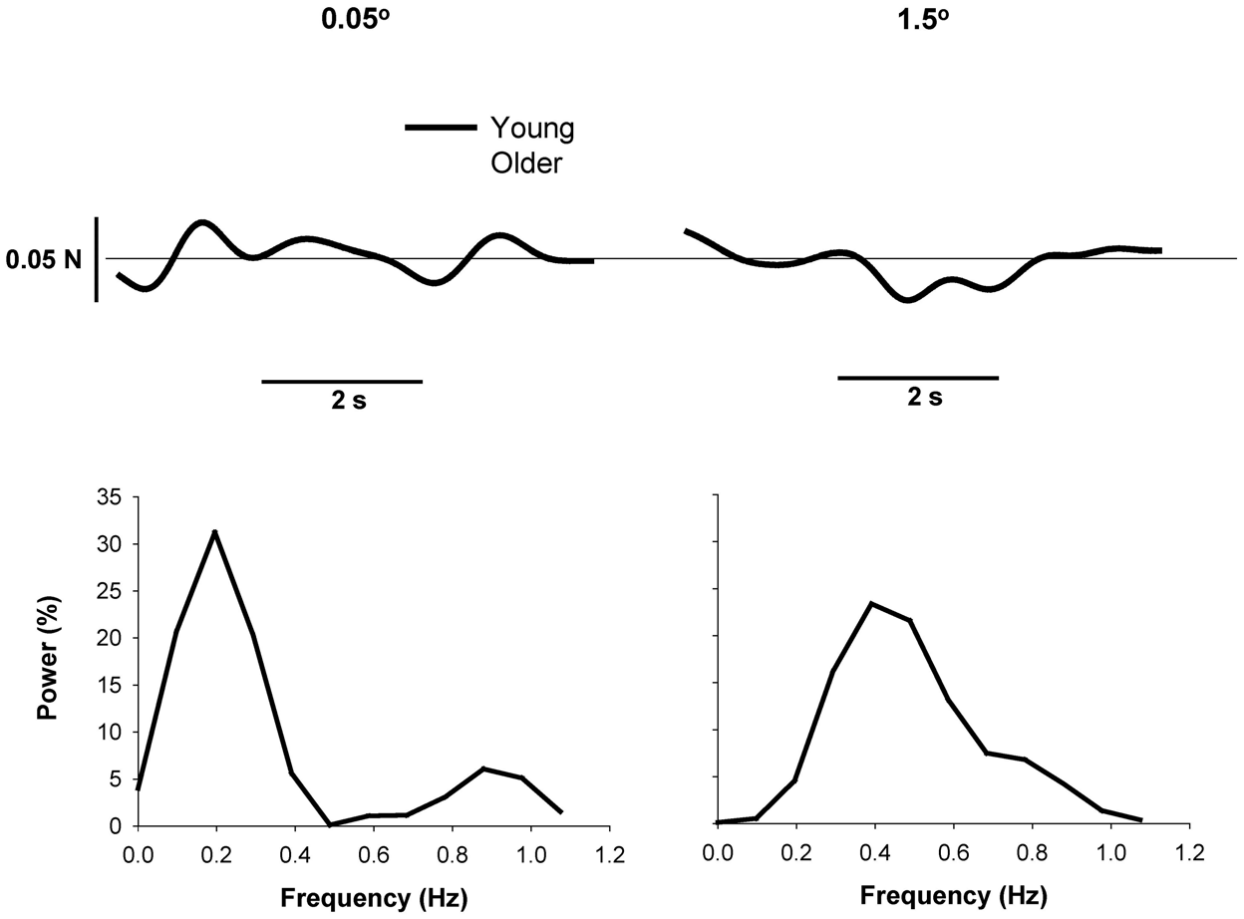
Modulation of power below 1 Hz for young and older adults with magnification of visual feedback. The top row shows a representative force output low-passed at 1 Hz for a young and an older adult during the lowest (0.05°) and highest (1.5°) visual angle. The bottom row demonstrates the power spectrum of force from 0–1 Hz for the same subjects. Age-associated differences in the modulation of force oscillations below 1 Hz are evident.

**Figure 7:**
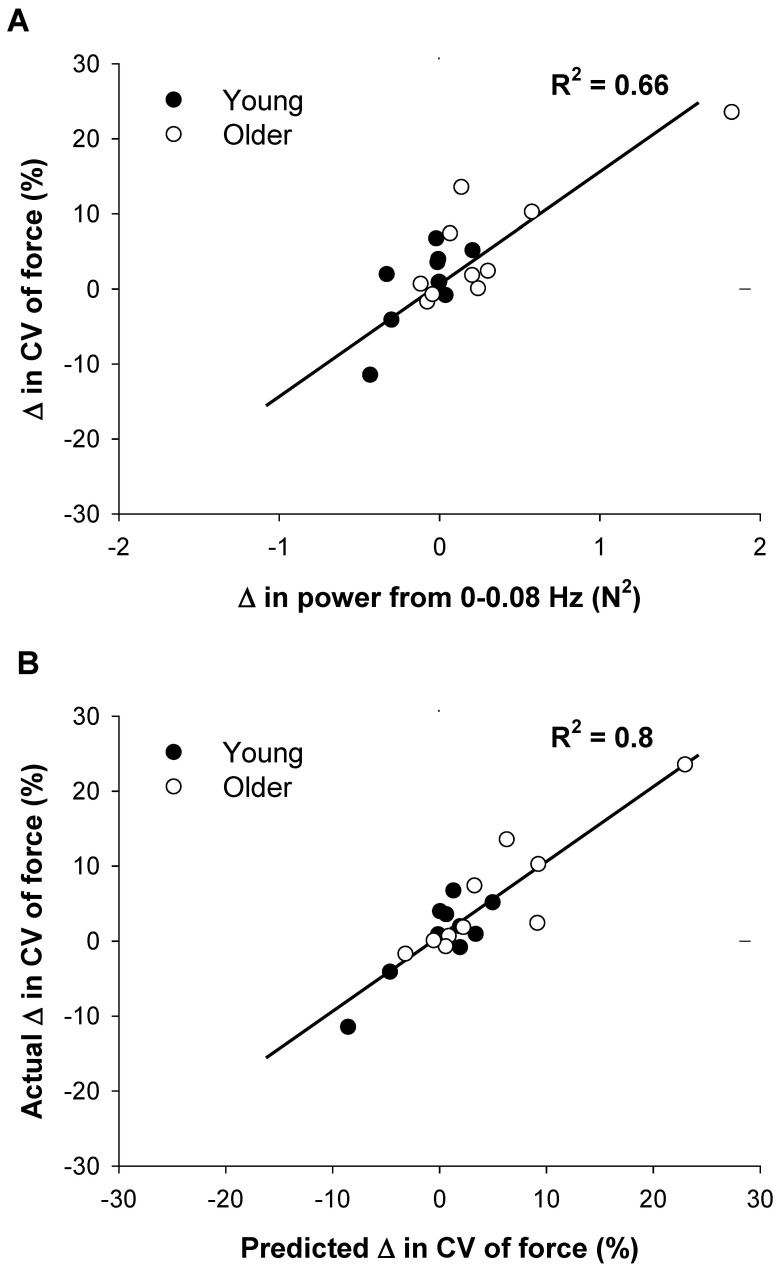
The association between modulation of force below 1 Hz and changes in the variability of force with magnification of visual feedback. A: For the absolute power spectrum, the change in the CV of force at the highest visual angle was associated with a decrease in absolute power from 0–0.08 Hz (R^2^ = 0.66). B: For the normalized power spectrum, modulation of force oscillations in 0.16 Hz, 0.33 Hz, 0.5 Hz, 0.66 Hz, and 0.83 Hz predicted changes in the CV of force at the highest visual angle (1.5°). The subjects (primarily older adults) who exhibited the greatest changes in CV of force at 1.5° visual angle increased power at 0.16 Hz, 0.5 Hz, and 0.83 Hz and decreased power at 0.33 Hz and 0.66 Hz.

We examined how the modulation of force oscillations below 1 Hz in young and older adults contributed to the increase in the variability of force with magnification of visual feedback. Specifically, we used a regression model to predict the changes in the CV of force from no visual feedback to the highest visual angle from the changes in absolute and relative power in specific frequencies below 1 Hz. We used the highest visual angle (1.5°) because age-associated differences in the variability of force were the greatest at this angle (Figure 2). Furthermore, the modulation of force below 1 Hz is different for young and older adults (Figure 5). These age-associated differences appear to be exacerbated from the lowest to the highest amount of visual feedback. As is evident from the representative sample in Figure 6, the variability of force did not change with magnification of visual feedback for the young adults but it increased substantially for the older adults. In addition, the absolute and relative power of frequencies below 1 Hz appears to be different for the two age groups (Figure 5). For the absolute power spectrum, the change in CV of force at the highest visual angle was predicted (R^2^ = 0.68, adjusted R^2^ = 0.66; P<0.001; Figure 7A) by a multiple-regression model that included the absolute power from the 0–0.08 Hz bin only. This regression model suggests that a reduction in CV of force with magnification of visual feedback was associated with a decrease in absolute power from 0–0.08 Hz. For the normalized power spectrum, the change in CV of force at the highest visual angle was predicted (R^2^ = 0.8, adjusted R^2^ = 0.73; P<0.05; Figure 7B) by a multiple-regression model that included the normalized power at 0.16 Hz, 0.33 Hz, 0.5 Hz, 0.66 Hz, and 0.83 Hz. This regression model suggests that a greater change in CV of force with magnification of visual feedback was associated with greater relative power at 0.16 Hz (part r = 0.43), 0.5 Hz (part r = 0.49), and 0.83 Hz (part r = 0.48), and lesser relative power at 0.33 Hz (part r = −0.48) and 0.66 Hz (part r = −0.45).

## Discussion

Age-associated differences in force control are evident in low-frequency force oscillations from 0–1 [4], [14] Hz and these low-frequency oscillations are altered when visual feedback is manipulated [15], [16]. Oscillations below 1 Hz have been shown in the activity of the motor cortex [20] and muscle [19] suggesting that slow oscillating signals may be detectable in force recordings and potentially provide insight into force impairments associated with aging. Thus, the purpose of this follow-up investigation was to determine whether a differential modulation of force below 1 Hz contributes to changes in force control related to aging and manipulation of visual feedback. We examined both the absolute and relative changes in power in 7 frequency bins. Our analysis of the normalized data, which reflects relative changes in the overall structure of the force signal (0–1 Hz), suggests that examining force oscillations below 1 Hz is important for understanding the interactive effects of aging and visual feedback on force control. When we removed visual feedback, subjects demonstrated an increase in the relative power in force oscillations from 0–0.33 Hz and decreased relative power from 0.66–1.0 Hz. In parallel to this finding, magnification of visual feedback decreased the relative power in force oscillations from 0–0.16 Hz and increased relative power from 0.66–1.0 Hz. Furthermore, our results indicate that older adults modulate these oscillations differently than young adults. Older adults exhibited greater normalized power from 0–0.16 Hz and lesser power from 0.66–0.83 Hz compared with young adults. This differential modulation of oscillations in force for young and older adults accounted for the age-associated differences in force control, especially with magnification of visual feedback. Manipulation of visual feedback and force oscillations below 1 Hz.

We examined the effects of visual feedback either by removing it or changing the visual feedback angle. When we removed visual feedback the normalized power from 0–0.33 Hz increased and power from 0.66–1.0 Hz decreased (Figure 3). Furthermore, when we magnified visual feedback by increasing the visual angle, the normalized power from 0–0.16 Hz decreased and normalized power from 0.66–1.0 Hz increased (Figure 4). Therefore, on average, our results indicate that magnification of visual feedback reduces the normalized power from 0–0.33 Hz and increases the normalized power from 0.66–1.0 Hz. This finding is intriguing because the normalized oscillations from 0–0.33 Hz are the greatest in the force output and contribute substantially to the variability of force. In order to control force (reduce variability of force), therefore, subjects must constrain the oscillations from 0–0.33 Hz. The magnified feedback may provide the opportunity to modulate these oscillations and improve force control. This proposition is supported by previous findings, which indicate that when visual feedback is magnified young adults reduce the fluctuations in force associated with increased breathing amplitude [8].

### Aging and Force Oscillations below 1 Hz

In this study, we provide evidence that older adults modulate force oscillations below 1 Hz differently than young adults. Under all conditions, older adults exhibited greater normalized power from 0–0.16 Hz and lesser power from 0.66–0.83 Hz compared with young adults (Figure 5). Additionally, our results indicate that the altered modulation of force oscillations below 1 Hz is associated with the greater age-associated differences in force control from the lowest to the highest amount of visual feedback. The greater change in CV of force at the highest visual angle for older adults (Figure 2) was predicted by a multiple-regression model that included the modulation of absolute power in the lowest frequency bin 0–0.08 Hz (R^2^ = 0.68; Figure 7A) as well as a model that included the modulation of normalized power at 0.16 Hz, 0.33 Hz, 0.5 Hz, 0.66 Hz, and 0.83 Hz(R^2^ = 0.8; Figure 7B). Interestingly, the modulation across these frequencies was not uniform. For example, an increase in the CV of force was associated with greater normalized power at 0.16 Hz (part r = 0.43) and lower normalized power at 0.66 Hz (part r = −0.45).

Modulation of slow oscillations in force may be particularly relevant to understanding why older adults exhibit impairments in performing activities of daily living. Impaired functional performance in older adults is associated with greater motor output variability. For example, practice-induced reductions in motor output variability for the index finger resulted in greater manual dexterity in older adults [37]. Furthermore, there is evidence that older adults exhibit greater postural sway than young adults because they exhibit greater motor output variability with the plantar flexor muscles [38]. In this study, we demonstrate that the amplification of force variability exhibited by older adults is associated with their inability to decrease force oscillations from 0–0.33 Hz.

### Potential Physiological Explanations

The focus of this study was to understand behavioral differences in force control below 1 Hz in young and older adults. This study, however, did not examine the physiological mechanisms associated with these behavioral findings. Future experiments should address the following questions to provide greater understanding of the mechanisms associated the modulation of force oscillations below 1 Hz and the exacerbation of the age-associated difference in force control with magnified visual feedback.

*Why do older adults exhibit greater relative power from 0–0.16 Hz compared with young adults under all visual feedback conditions?* The increased relative power in force oscillations from 0–0.16 Hz for older adults may be due to greater synaptic noise that results in greater discharge rate variability of motor units. There is substantial evidence that older adults exhibit greater discharge rate variability compared with young adults during a variety of motor tasks [23], [37], [39], [40]. The association between synaptic noise and discharge rate variability of motor units has been shown through experimental studies [41], [42] as well as computer simulations [43] and is most evident during low-force contractions. The variability of motor unit discharge, furthermore, is associated with low frequency modulation of the motor neuron pool [29], [44]. Moreover, coherence at low frequencies is stronger in older adults suggesting a fundamental difference in oscillatory inputs to motor neurons [45].

*Why does magnification of visual feedback exacerbate the age-associated differences in force control?* Our results suggest that alterations in visuomotor integration may underlie the age-associated differences in force modulation with magnification of visual feedback. Numerous neuroanatomic structures and physiologic processes are sensitive to aging and, therefore, may underscore age-related changes in visuomotor integration [46], [47], [48], [49], [50]. For instance, when visual feedback is magnified during a precision grip task, select regions within the parietal and premotor cortices demonstrate increased activation [46]. Thus, the deficits exhibited by older adults could be explained by cortical activation changes in these specific areas.

Magnification of visual feedback not only increases the demand for cortical resources associated with visual processing, but also increases the attentional requirements of the task. An impaired ability to activate cortical regions associated with attentional control, therefore, may be another reason why older adults were unable to modulate force output with magnified visual feedback as well as young adults [47], [48]. Finally, magnification of visual feedback may increase the stress associated with task performance [49], [50], which has been shown to increase low frequency force fluctuations in older adults [1], [39].

### Methodological Considerations

In this follow-up investigation, we studied the modulation of force oscillations below 1 Hz by examining the absolute and relative power in 7 frequency bins. We used both analyses to provide a more comprehensive examination of slow oscillating force signals. The absolute power analysis is influenced by the amplitude of force variability and thus, better reflects the changes in force variability associated with the aging and visual feedback manipulations (see Figure 3A). In contrast, the relative power analysis is independent of the amplitude of force variability and thus, better reflects the overall structure of the force signal within the band of interest. Our results from these two analyses suggest that the normalized analysis may be more sensitive to identify age differences in force modulation as a function of changes in visual feedback. One explanation for this is that with normalization of the power within 1 Hz, the 7 frequency bins are co-dependent on eachother and are not influenced by changes in power at higher frequencies (>1 Hz). Interestingly, despite the numerous ways that power can be redistributed among the 7 frequency bins, aging and manipulation of visual feedback consistently modulated power primarily in two frequency bins.

In summary, our results indicate that modulation of force oscillations below 1 Hz is related to changes in force control associated with manipulation of visual feedback and aging. Magnification of the visual feedback decreased the relative proportion of force oscillations from 0–0.33 Hz and increased relative force oscillations from 0.66–1.0 Hz. Furthermore, we demonstrate a differential modulation of frequencies below 1 Hz for young and older adults, which was associated with the exacerbation of age-associated differences in force control when visual feedback was magnified. Overall, our findings suggeste that understanding force oscillations below 1 Hz is important for determining the effects of visual feedback and aging on force control. Future research should further examine the oscillations in force below 1 Hz and identify the neural mechanisms that contribute to these oscillations and their functional implications.
